# Three-Dimensional Structure of a Simple Liquid at a Face-Centered-Cubic (001) Solid Surface Interface

**DOI:** 10.1038/srep29786

**Published:** 2016-07-19

**Authors:** Luyao Bao, Haibao Hu, Jun Wen, Paavo Sepri, Kai Luo

**Affiliations:** 1 School of Marine Science and Technology, Northwestern Polytechnical University, Xi’an, Shaanxi 710072, Peoples R. China; 2 College of Engineering, Florida Institute of Technology, Melbourne, Florida 32901, USA

## Abstract

A liquid in the vicinity of a solid-liquid interface (SLI) may exhibit complex structures. In this study, we used molecular dynamics simulations demonstrating for the first time that the liquid adjacent to the SLI can have a two-level structure in some cases: a major structure and a minor structure. Through a time-averaging process of molecular motions, we identified the type of the liquid structure by calculating positions of the maximum liquid density in three spatial dimensions, and these positions were found to distribute in many dispersed zones (called high-density zones (HDZs)). The major structure appears throughout the SLI, while the minor structure only occurs significantly within the third layer. Instead of the previously reported body-centered cubic (BCC) or face-centered-cubic (FCC) types, the major structure was found to show a body-centered tetragonal (BCT) type. The adjacent HDZs are connected by specific junctions, demonstrating that atoms diffuse along some particular high probability paths from one HDZ to another. By considering the three-dimensional liquid density distribution from the continuum point of view, more complete details of the structure and diffusive behavior of liquids in the SLI are also possible to be revealed.

In order to better understand phenomena such as wetting, segregation, crystal growth, and perhaps applied topics such as Kapitza resistance and slip^1^,^2^,^3^,^4^,^5^,^6^,^7^,^8^, it is important to investigate issues of fundamental importance at the molecular level. Some of these issues involve the identification of the structural order type exhibited by a liquid in contact with a flat crystal surface. Will this structure be a simple body-centered cubic (BCC) or face-centered-cubic (FCC)^9^,^10^? Will the atoms in the solid-liquid interface (SLI) equally likely to go in all directions when they undergo intermittent hopping^11^, or are there preferred directions?

The degree of order within a liquid is higher near a solid surface than is exhibited in the short-range order in bulk liquids, which has been observed in both theoretical and experimental investigations^6^,^12^,^13^,^14^,^15^,^16^,^17^. Recently, main results of ordered structures of a liquid in the SLI include two aspects: one is density fluctuations perpendicular to the interface, called the layering order, and the other is in-plane order, which describes the order of liquid occurring parallel to the SLI. The layering order has been well studied by experiments^4^,^18^,^19^,^20^,^21^,^22^ and atomistic simulations^7^,^23^,^24^,^25^. These works have consistently demonstrated that the layering order is universal in the SLI, and its features are sensitive to its environment and properties of both the solid substrate and the bulk liquid.

The in-plane order has been observed experimentally^1^, and the results are consistent with earlier molecular dynamics (MD) simulations^1^. In addition to experimental revealing, research could also benefit from alternative techniques, atomistic simulations, in obtaining detailed data on the atom positions in the SLI. Atomistic simulations are able to provide atom-resolved insights into phenomena occurring near the interface^26^. Molecular dynamics simulations^15^,^23^,^27^ indicate that the liquid can form several different structures (such as BCC or FCC) under different conditions, and that the structural type may differ with different solid substrates, or if the liquid condenses to the solid.

Some researchers have described the ordering of liquid structures near the interface being similar to that of a pure solid from the atomistic point of view^10^. Although this appears to work for the first few layers, liquid-like features become dominant, as the distance to the interface increases. Other researchers^6^,^7^,^9^,^23^,^27^,^28^ have concentrated on the liquid structure using a statistical approach. Some have investigated the time-averaged atomistic positions in two-dimensional planes parallel to the solid surface. Such two-dimensional representations may miss important features of liquid structures between adjacent planes.

In the present study, we extended our molecular dynamics computations to include the structure of the liquid in three dimensions. Structures and behaviors of liquid argon in contact with a platinum FCC (001) crystal surface are studied. We found that the liquid has a two-level structure, and that liquid atoms near the SLI hop along two paths of high probability. We also discuss the mechanism of the formation of liquid structure in the SLI based on the Boltzmann law.

## Methods

**Figure 1:**
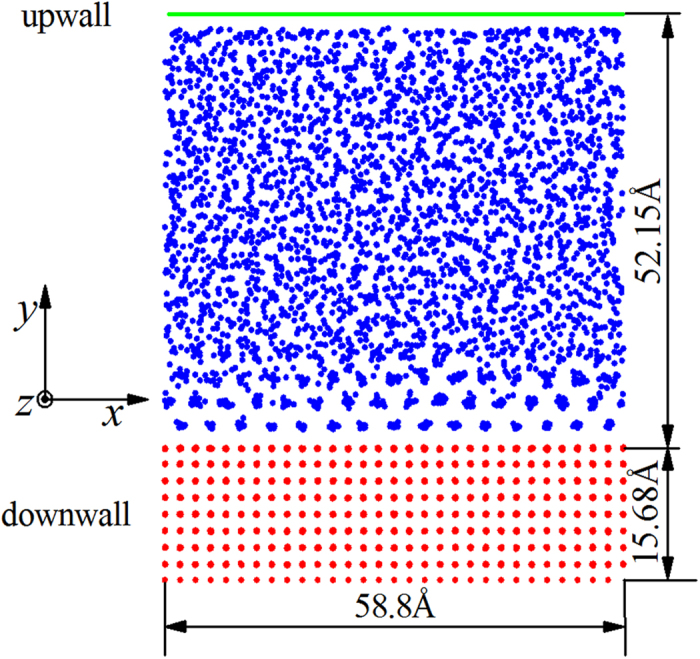
Front view snapshot of the molecular dynamics simulation. The red points are solid atoms in the lower wall and the blue points are liquid atoms. The green line represents a structureless Lennard-Jones (LJ) flat upper wall, which constrains the liquid atoms.

We focused on the liquid argon confined between an FCC (001) solid platinum bottom wall and a numerical top wall, as shown in Fig. 1. The *x*, *y*, *z* dimensions of the simulation region are (−29.4, +29.4), (−58.0, +10.0), (−29.4, +29.4), respectively, whose units are angstroms (Å). The linear extent of the *y* direction is sufficient to accommodate the existence of some weak liquid layers in the central part of the simulation box, indicating that the liquid is confined. These system dimensions were chosen to facilitate comparison to the cases where the liquid is completely unconfined.

The interaction between atoms in the simulations is taken to be the Lennard-Jones 12-6 potential function (LJ/12-6 potential): *U*(*r*) = 4*ε*[(*σ*/*r*)^12^− (*σ*/*r*)^6^]. Here, *r* is the distance between two argon atoms, *ε* is the potential well depth, and *σ* is the distance of zero potential. The Lennard-Jones parameters for argon are: *ε* = 0.01042 eV, *σ* = 3.405 Å, and the mass is: *m* = 39.948 g/mol. A cutoff distance (*r*_*c*_ = 2.5*σ*) for the LJ/12-6 potential is adopted to improve the computational efficiency. The interaction between two atoms is considered to vanish when the distance between them is larger than *r*_*c*_. We chose the LJ/12-6 potential to describe the liquid argon because this potential has been shown to replicate well the thermodynamic properties of argon obtained experimentally.

The equilibrium temperature and bulk number density of the liquid argon are taken to be 85 K and 0.0196 Å^−3^, respectively. A noteworthy observation in Fig. 1 is that liquid atoms in the first and second rows are instantaneously positioned within alternating cells of spacing 3.92 Å. The view seems to show clustering of atoms in these cells, but that is merely a superposition of many atoms along the *z* direction.

At this point, we provide some more information concerning the thermodynamic state of the liquid argon. Johnson *et al*.^29^ improved on the Nicolas^30^ equation of state for LJ liquids by using new molecular dynamics simulations. He arrived at a new set of parameters for the 33-parameter modified Benedict-Webb-Rubin (MBWR) equation. According to the reported state parameters for liquid argon and the MBWR equation of Johnson *et al*., the thermodynamic state of the liquid argon in our case lies in the phase diagram region between the coexistence and spinodal curves^31^, but does not lie close to either curve. Due to the confinement, the phase diagram of liquid argon will be slightly different from that of the bulk argon^32^. Therefore, it is reasonable to assume that the thermodynamic state of argon here is in the above-mentioned region of the phase diagram.

The bottom wall consists of nine layers of platinum FCC (001) crystal and contains 4050 atoms (the red points in Fig. 1). Note that face-centered atoms are also shown without distinction from the corner atoms. The lattice parameter is denoted by *a* (3.92 Å), and the solid is described by the potential of the embedded-atom method (EAM)^33^. The spacing of red atoms in Fig. 1 is thus 1.96 Å. The cutoff distance, mass of solid atoms, and the tabulated values of the functions are taken from S. M. Foiles *et al*.^33^. The top wall (the green line in Fig. 1) is modelled to be a flat LJ/12-6 potential boundary.

Interactions between solid and liquid atoms play an important role in simulating the liquid structure at the SLI. We chose the LJ/12-6 potential to describe these interactions, as other investigators have successfully applied it to the same topic^10^,^23^,^34^, and even other interfacial phenomena, such as wetting^35^,^36^,^37^ and nanofluidics^38^,^39^,^40^ using MD simulations. This potential is less satisfactory to model the liquid-solid interaction especially for experimentalists. However, our paper focus on the general trends of local order rather than attempting to extract physically meaningful numbers^2^. And the general trends from such simulations are important for understanding the general phenomenon^2^.

The depth of the LJ/12-6 potential, denoted by *ε*_Solid-Liquid_ (*ε*_SL_), can be used to represent the wettability of the liquid to the solid^41^. For example, *ε*_SL_ is set equal to *ε*_liquid-Liquid_ (*ε*_LL_), indicating that the solid substrate is very hydrophilic to the liquid^40^,^42^. Under this circumstance, the platinum crystal has no particular influence other than providing an atomistic solid substrate. Different lattice structures of the solid substrates should generate different liquid structures at the SLI owing to the change in cell size of the potential field configuration^43^. However, such variations are not the focus of our present work. In all cases, the distance of zero potential between platinum atoms is 2.935 Å, which is used to determine the potential parameter (*σ*) between platinum and argon atoms using the Lorentz-Berthelot mixing rule.

An initial period of computation is completed first in order to achieve an equilibrium state as follows. Solid atoms are arranged within the bottom wall in an FCC crystal structure (*a* = 3.92 Å), and the liquid atoms are arranged internally in an FCC structure with a lattice parameter of 1.34*a*. The temperature of the liquid is then increased to 2000 K to produce complete disorder in the liquid^10^. The positions of solid atoms are kept unchanged in this process. After the liquid reaches an initial equilibrium, the liquid is then cooled to 85 K, and the temperature of the bottom wall is set at 85 K to simulate a flexible wall. The thermostat method used in all these processes is that of Nosé-Hoover. After the system reaches this second equilibrium, the thermostat is removed to make the system into an NVE ensemble. All the simulations were carried out using the LAMMPS code^44^ with a timestep of 0.002 ps. The three spatial dimensions (*x*, *y*, *z*) are defined such that *y* is the perpendicular distance away from the wall. Periodic boundary conditions are imposed at the extremities of both *x* and *z* directions for solid and liquid alike. Whenever an atom crosses such a boundary, it will renter into the simulation box at the opposite boundary. While the atoms move in a random thermal sense, there is no macroscopic cross-flow imposed either in the *x* or *z* directions.

The influence of periodic boundary conditions on the thermodynamic state of system becomes insignificant in a statistical sense as the system exceeds certain spatial dimensions. In order to verify this, we have run four simulations with argon to observe the effect on the pressure at different temperatures in the range 85–115 K. The first simulation, (a), was with the same dimensions as the simulation reported herein. The other simulations were with dimensions larger than the first: (b) double the first simulation in the *x* direction; (c) double the first simulation in the *z* direction; and (d) double the first simulation in both *x* and *z* directions. The resulting average pressures of argon differed insignificantly among these simulations. Therefore, we conclude that the sizes used in the simulation reported here are sufficiently large to model the bulk liquid argon.

The three-dimensional volume is divided into an equal mesh with spacings: Δ*x* = 0.392 Å, Δ*y* = 0.4 Å, and Δ*z* = 0.392 Å. The time-averaged density distribution, *ρ*(*x*, *y*, *z*), is calculated at corresponding cells. To help with the analysis, *ρ*(*y*) is also calculated by time averaging the number density of atoms in a slice of width Δ*y* = 0.01 Å parallel to the solid surface.

## Results

We first investigated the three-dimensional liquid structure at the SLI for two different cases, namely: (1) a high degree of incommensurability in size between solid and liquid atoms^40^,^43^, and (2) a very hydrophilic substrate.

The layering order is characterized by the decaying oscillation of the liquid density (denoted as *ρ*(*y*) in this paper) perpendicular to wall. In this sense, *ρ*(*y*) is considered to be a continuous function, and it represents both the solid-like and liquid-like features of the liquid itself in the SLI. We extended this computational method to three dimensions, so that the time-averaging procedure produced a probabilistic function of the liquid density distribution in space, *ρ*(*x*, *y*, *z*). Here, the density at each location represents the total mass-centers of atoms per unit volume statistically. We are able to identify both the fluid structure and atom mobility using *ρ*(*x*, *y*, *z*). For the bulk crystal without any lattice faults, the three-dimensional fluid density consists of many dispersed spheres whose centers congregate within the potential function lattice cells generated by the solid atoms oscillating around their lattice points. We can identify the structure near the SLI based on the positions of the density spheres, and we can measure the atom mobility quantitatively from the dynamic details of the spheres. For the bulk region of the liquid, *ρ*(*x*, *y*, *z*) is uniform in space because atoms are able to diffuse in all directions without bias.

**Figure 2:**
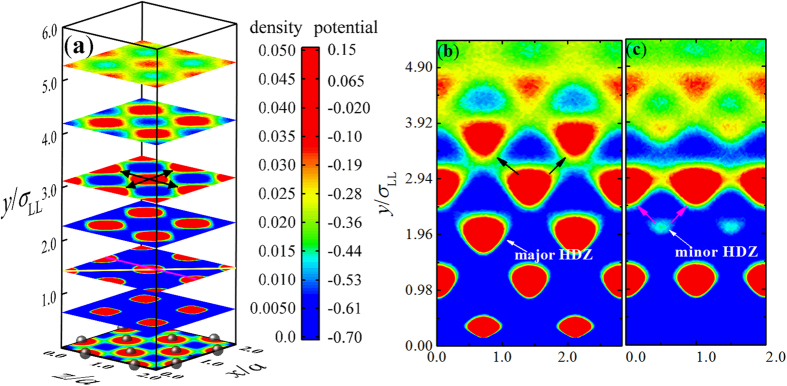
(**a**) The top six planes show the two-dimensional liquid density, (*ρ*(*x*, *y*_0_, *z*), parallel to the wall at the specified locations, *y*_0_, perpendicular to the wall. These six locations correspond to the first six maxima of *ρ*(*y*). *a* is the lattice constant of lower wall. The brown solid spheres reside in the first layer of the solid wall and the bottom plane depicts the wall-potential at the location of the second plane. We only show segments of the horizontal planes to make the density distribution clear. The density distribution is shown in the elevated planes with corresponding periodicities and amplitudes. (**b**,**c**) represent vertical cuts of liquid density obtained by intersections through the third plane in (**a**) at the yellow and pink solid lines, respectively. The black solid arrows in (**a**,**b**) show the high-probability hopping paths for liquid atoms within the major structure. The magenta solid arrows in (**c**) show the high-probability hopping paths within the minor structure. The time span used for averaging is 200 ns, which is long enough to obtain a smooth distribution for *ρ*(*x*, *y*, *z*).

We define the boundaries of liquid layers parallel to the wall where the density distribution, *ρ*(*y*), achieves successive minima. These planes appear predominantly blue in color as surmised from Fig. 2(b,c). The planes shown in Fig. 2(a) represent cuts through the central portions of the liquid layers. The first layer is comprised of the liquid between the wall and first minimum of *ρ*(*y*). We examined the first six layers and denoted them as *Nos*. 1~6 successively away from the wall. The liquid is clearly distributed into periodic cells of high density, which we call high-density zones (HDZs). Surprisingly, it appears that the liquid structure consists of two different types of HDZs rather than a single one. For the first type, the liquid density is high and the structure extends throughout the SLI region (Fig. 2). Therefore, we call these to comprise the major HDZs. For the second type, the liquid density is much lower than in major HDZs, and thus we call them the minor HDZs. The minor HDZs are shown in Fig. 2(c) within the third layer significantly, and perhaps in the fifth layer.

The centers of HDZs are defined to be the positions of liquid density maxima, analogous to the density of the bulk solid mentioned earlier. We define the liquid structure as the arrangement of the HDZs centers. The presence of two types of HDZs demonstrates that there is a two-level structure for the liquid in the SLI under the conditions of the present simulation. We named the arrangement of the major and minor HDZs the major and minor structure, respectively. Within the SLI region, the transition from high ordering at the interface to the bulk liquid further away appears to have this compound structure.

**Figure 3:**
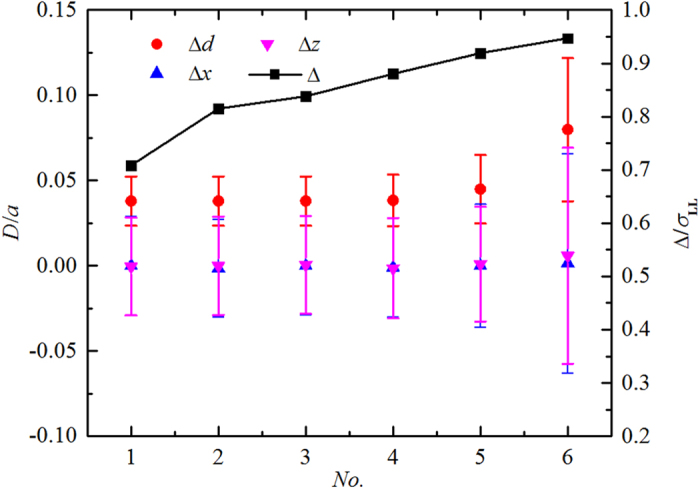
Quantitative evidence of the BCT arrangement of the centers of the major HDZs. *Nos* 1–6 are the first six layers successively away from the wall. The error bars are standard deviation from 210 HDZs for corresponding data.

Figure 2(a) indicates that the major structure of liquid is of BCC or BCT types. We confirm this by further analyzing the simulation results. There are 210 major HDZs for each layer in our simulation. First, we identify the positions (*x*_*i*_, *y*_*i*_, *z*_*i*_) of the centers of the major HDZs and compare the *x*_*i*_ and *z*_*i*_ components with the corresponding components *x*_*p_i*_ and *z*_*p_i*_ coming from the first layer at the lower wall. Three differences in location are calculated as: Δ*x*_*i*_ = *x*_*i*_−*x*_*p_i*_, Δ*z*_*i*_ = *z*_*i*_−*z*_*p_i*_ and Δ*d*_*i*_ = ((Δ*x*_*i*_)^2^ + (Δ*z*_*i*_)^2^)^1/2^. Then, we obtained the average and variance of these three quantities for the 210 HDZs in each horizontal layer. The results are shown in Fig. 3.

The value of Δ*d* (average of the individual Δ*d*_*i*_) for each layer is much less than the bottom-wall lattice constant *a.* The corresponding red solid circles in Fig. 3 demonstrate that the centers of the major HDZs line up in a vertical structure. In addition, the values of Δ*x* and Δ*z* (average of the Δ*x*_*i*_ and the Δ*z*_*i*_, respectively) are nearly zero, as expected (the blue and pink solid triangles in Fig. 3). The error bars show the range of variation in these measures. Another interesting observation is about the locations of the HDZ planes, *y*_*i*_, corresponding to the positions of the maxima of *ρ*(*y*) as mentioned in the caption of Fig. 2(a). The difference (Δ) between the values of *y*_*i*_ of adjacent layers increases as the layers get further from the bottom wall (the black solid squares in Fig. 3). This indicates that the separation between HDZ layers increases progressively away from the wall. These observations demonstrate that the major structure of the liquid in the SLI is BCT instead of BCC or FCC under conditions of the present simulation. The HDZs of the minor structure are also aligned vertically above and below the centers of their unit cell respectively (Fig. 2(c)).

The HDZs within the liquid result from the vibrational behavior of atoms similar to that of the solid, and this might be termed a solid-like feature of SLI. However, liquid atoms do not vibrate in the HDZs all the time, and will occasionally hop from one HDZ to another both horizontally and vertically. Such motion has been called the intermittent-hopping behavior^11^, and this could be considered a liquid-like feature of the SLI. The density distribution is proportional to the probability that liquid atoms appear at any given position. In Fig. 2(a–c), there are specific junctions that exist between adjacent HDZs, which demonstrate that hopping atoms follow particular high probability paths within one BCT unit cell. In the horizontal direction, atoms hop along the edges of the BCT unit cell (the black horizontal arrows in Fig. 2(a)). In the vertical direction, atoms follow two paths: one is along vertices towards the center of the BCT unit cell (the inclined black arrows in Fig. 2(b)); and the other is from a vertex of a minor HDZ and towards the adjoining major HDZs of the unit cell (the magenta arrows in Fig. 2(c)). We call the hopping paths in the first and second level structures the major and minor paths, respectively.

Geysermans^6^,^34^ *et al*. have also discussed the diffusion paths and in-plane order of the liquid at the SLI using a MD simulation. They proposed that the diffusion behavior of liquid atoms relaxes the misfit stresses existing in the liquid layer. In our present study, we have been able to supply additional details in three dimensions concerning two types of structures and hopping paths of the liquid at the SLI.

## Discussion

The density distribution in general be a direct result of the Boltzmann law which states that density is proportional to exp(−*U*/*k*_B_*T*), where *U* and *T* are the potential energy and temperature at some positions, and *k*_B_ is the Boltzmann constant. Atoms at the bottom wall generate low-potential wells (WLPWs) which arrange with periodicity parallel to the wall (the first plane in Fig. 2(a)). These WLPWs tend to trap liquid atoms and cause them to vibrate around the lowest points of the WLPWs, thereby resulting in the formation of the first liquid layer (FLL). The FLL will generate a similar potential field leading to the formation of the second liquid layer. Thus, the structure of the wall is transmitted into the liquid forming the major structure.

**Figure 4:**
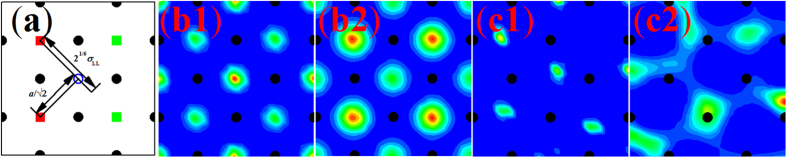
(**a**) Illustration of the lowest potential distance *L*_1_ = 2^1/6^*σ*_LL_ from the reference liquid atom (solid squares) and the distance *L*_2_ = *a*/√2 from the lowest potential generated by four nearest solid atoms (solid circles) to the same reference liquid atom. *a* is the lattice constant of the solid; The in-plane order of the first (b1,c1) and second (b2,c2) layers: (**b**) *ε*_SL_ = ε_LL_ and *σ*_SL_ = 2.954 Å, unconfined; (**c**) *ε*_SL_ = 7*ε*_LL_ and *σ*_SL_ = 2.954 Å, unconfined. The time span used for averaging is 200 ns which is long enough to obtain the smooth *ρ*(*x*, *y*, *z*).

The depth in the sequence of low-potential wells (LPWs) gets progressively shallower away from the wall. This can be attributed to the increasingly diffusive behavior of atoms evidenced by the change in connections between adjacent HDZs. In Fig. 4, we show the relationship of the HDZ locations relative to two characteristic length scales: *L*_1_ = 2^1/6^*σ*_LL_ and *L*_2_ = *a*/√2. The former represents the distance to the minimum point of the LJ/12-6 potential, and the latter represents the diagonal length in the crystal lattice structure. In Fig. 4(a), the solid squares represent the locations of the HDZs in the third layer, and the solid circles represent the locations of solid atoms at the wall. Therefore, at the third layer the depth of the LPWs generated by the atoms in the two nearest major HDZs (the two red solid squares in Fig. 4(a)) are shallower but comparable to the depth of LPWs generated by the atoms in all four nearest major HDZs (the four solid squares in Fig. 4(a)) in this simulation. Some liquid atoms may appear in the LPWs generated by the two nearest major HDZs at a lower probability than the atoms in the major HDZs. Thereby, this may explain the appearance of the second level structure of liquid in the SLI. The same argument does not apply for the first two liquid layers shown in Fig. 2 according to our simulation results.

The configuration of the FLL determines the entire structure of the SLI according to the previous analysis. Therefore, we only need to discuss the formation of the FLL. If one atom in the major HDZ tries to enter into an empty region of the WLPZ to change the configuration of the FLL, the adjacent HDZs will impose a repulsive force to prevent this from happening since *L*_1_ is larger than *L*_2_ (Fig. 4(a)). The intensity of this prevention depends on the probability of atoms appearing in the four nearest major HDZs. This probability depends on the number of atoms occupying the FLL. In our simulation, the time-averaged number of atoms in the FLL is 12.4377, which means that the probability of the WLPZ being occupied by atoms is 0.05923 (divide the above by 210, the total number of WLPZs). It turns out that this probability is large enough to maintain the configuration of the FLL as shown in Fig. 2(a). From the point of view of the Boltzmann law, the potential generated by the four nearest HDZs counteracts the depth of the WLPZs located at these HDZs statistically. Therefore, the probability that liquid atoms are trapped into these WLPZs is so low that the liquid atoms prefer to enter into other deeper LPZs generated by the FLL or remaining liquid layers. Thus, the configuration of the FLL is maintained.

We verify this assertion by carrying out another simulation where the top wall is 10 Å higher than previous cases in order to create a free liquid-gas interface. Under this condition, the time-averaged number of atoms in the FLL reduces to 11.0216 and the probability of a WLPZ occupied by atoms reduces to 0.05248. As expected, all the WLPZs are occupied and the HDZs appearing now are weaker than their four surroundings (Fig. 4(b1)) owing to the offset of these WLPZs from atoms in the surrounding four strong HDZs. In addition, the HDZs in the successive layers arrange based on the first layer (Fig. 4(b2)). The structure in Fig. 4(b1,b2) indicates that the configuration of the two-level structure of liquid in the SLI is not unique but changes with the conditions of the system.

Another reason for the formation of the structure shown in Fig. 2 is the mobility of liquid atoms in the FLL, which also leads to similar HDZs in all layers. The solid-liquid interaction is equal to the liquid-liquid interaction. The liquid atoms in the FLL possess enough mobility to hop horizontally and vertically owing to the complete liquid state in the bulk liquid. Therefore, the LPZs each have an equal probability for occupation by atoms at any given time. Hence, the HDZs in every layer have the same structure. If we decrease the mobility of atoms in the FLL by increasing the strength of the wall potential, the HDZs in the first layer should become different from each other and the in-plane order might even disappear. The results of simulations shown in Fig. 4(c1,c2) are consistent with our explanations. Here, the solid-liquid interaction in case (c) is 7 times greater than that in case (b). This strength of interaction is approximately equal to the interaction between Pt and Ar atoms according to Lorentz-Berthelot mixing rule, if the interaction between Pt and Ar atoms is modeled by the LJ/12-6 potential. Under this condition, there is no periodic structure of liquid in the solid-liquid interface according to the third simulation results in our paper. As the high temperature leads to appropriate mobility of liquid atoms in the first liquid layer, the periodic structure including the minor structure is expected to exist in physical Pt/Ar system. In other word, the minor structure predicting from our simulations will exist in the system under (1) a high degree of incommensurability in size between solid and liquid atoms, and (2) a very hydrophilic substrate (*ε*_SL_ is approximately equal to *ε*_LL_). In addition, this result also demonstrates that the configuration of the FLL determines the entire structure of the SLI.

The slip phenomenon sometimes appearing in liquid-solid systems directly relates to the liquid structure at the SLI^39^,^45^. In the molecular kinetic theory of slip, slip is mainly caused by liquid atoms hopping from one equilibrium site to another, passing through a location of higher energy^46^,^47^. Also, the slip between liquid layers at the SLI makes a contribution to the apparent slip at high rates of shear^47^. Once we know the three-dimensional density distribution of flowing liquid at the SLI, we will be able to obtain the rate of liquid atoms hopping from one site to another directly according to the molecular kinetic theory of slip^47^. The momentum transport between the liquid layer at the SLI is more complicated due to the unknown but significant^48^ influence of liquid structure order on viscosity in sequential layers. Despite of this, it is of great value to analyze the stress tensor by referring to the three-dimensional density distribution.

The implications for segregation have been well commented by Kaplan and Kauffmann^2^. According to their argument, the concept of a decrease in entropy due to ordering should be taken into account for segregation at solid-liquid interfaces, where segregation and ordering both occur. To this end, the complete three-dimensional liquid structure should be helpful in describing the combined ordering-segregation phenomenon using the framework of the multilayer adsorption^2^.

## Additional Information

**How to cite this article**: Bao, L. *et al*. Three-Dimensional Structure of a Simple Liquid at a Face-Centered-Cubic (001) Solid Surface Interface. *Sci. Rep.* **6**, 29786; doi: 10.1038/srep29786 (2016).
